# Bionic Design for Mars Sampling Scoop Inspired by Himalayan Marmot Claw

**DOI:** 10.1155/2016/5713683

**Published:** 2016-12-29

**Authors:** Long Xue, Rong Rong Zhang, Wei Zong, Jia Feng Song, Meng Zou

**Affiliations:** ^1^Key Laboratory of Bionic Engineering, Ministry of Education, Jilin University, Changchun 130022, China; ^2^School of Mechatronics and Engineering, East China Jiaotong University, Nanchang 330045, China; ^3^Aerospace System Engineering Shanghai, Shanghai 201108, China

## Abstract

Cave animals are often adapted to digging and life underground, with claw toes similar in structure and function to a sampling scoop. In this paper, the clawed toes of the Himalayan marmot were selected as a biological prototype for bionic research. Based on geometric parameter optimization of the clawed toes, a bionic sampling scoop for use on Mars was designed. Using a 3D laser scanner, the point cloud data of the second front claw toe was acquired. Parametric equations and contour curves for the claw were then built with cubic polynomial fitting. We obtained 18 characteristic curve equations for the internal and external contours of the claw. A bionic sampling scoop was designed according to the structural parameters of* Curiosity*'s sampling shovel and the contours of the Himalayan marmot's claw. Verifying test results showed that when the penetration angle was 45° and the sampling speed was 0.33 r/min, the bionic sampling scoops' resistance torque was 49.6% less than that of the prototype sampling scoop. When the penetration angle was 60° and the sampling speed was 0.22 r/min, the resistance torque of the bionic sampling scoop was 28.8% lower than that of the prototype sampling scoop.

## 1. Introduction

Being the most similar planet to Earth in the solar system, Mars is an important focus of deep space exploration [1, 2]. Analyzing Martian soil can help us to discover whether there is water, or even life, there and provide answers to other key questions, but the surface of Mars is complex, and its shallow soil can be frozen and stony [3]. The uneven surface leads to irregular changes in resistance and torque during sampling, which affects the energy consumption and stability and efficiency of the process. In addition, mass and space limitations at launch create many design and optimization challenges.

The scoop on the* Viking* spacecraft launched by NASA in 1975 had a sampling depth of 10 cm and energy consumption for soil samples of 1 cm^3^ of 4–9 kJ [4, 5]. In 2008, NASA's* Phoenix* Mars lander had a multifunction sampling shovel that could not only perform a digging action to make a hole but also obtain samples from below the surface soil by means of a tip and serrated blade at the end of the shovel [6, 7]. In 2011, NASA launched* Curiosity*, a Mars patroller equipped with a sampling shovel able to scoop, sieve, and portion samples of powdered rock and soil [8]. A pliers-type sampling mechanism developed by the Hong Kong Polytechnic University was used on the* Beagle 2* Mars mission of the European Space Agency (ESA) [9, 10].

In the course of research on Mars sampling devices and the shape of the sampler, Johnson and King studied horizontal and vertical stress distribution during sampling with a bucket-wheel sampler digging simulative Martian soil [6]. Craft et al. demonstrated a percussive digging system to decrease the downforce needed to penetrate a given soil [8]. Based on the European* Beagle 2* lander, Richter et al. developed a device that could conduct shallow surface sampling and collect samples from about 0.5 m below the surface [11]. Gruntz developed a grab sampler that can be used for grabbing samples of soil or rock [12]. Kubota et al. developed an end effector that can dig samples and showed good stability after test certification [13].

In nature, many cave animals have evolved clawed toes with mechanical properties that help reduce resistance. The Himalayan marmot, which lives in the Qinghai-Tibet plateau, is an example of a soil-burrowing animal adapted to climate, soil, and other conditions of its environment [14, 15]. Its remarkable digging performance is the inspiration for the design of a new sampler for alien planets. Methods of bionic structural engineering are used to research different biological structures based on prototypes from nature and mechanical principles, and then bionic simulations of the engineering structures are used to enhance the efficiency of the external form and internal mechanisms.

Taking the Himalayan marmot's second front clawed toe as the prototype, the configuration optimization of the sampler was studied through theoretical analysis and simulation to reduce resistance during digging. The practicability and effectiveness of this method of bionic design and optimization were then verified by experiments.

## 2. Biological Prototype

### 2.1. The Geometry of the Himalayan Marmot's Clawed Toe

**Figure 1:**
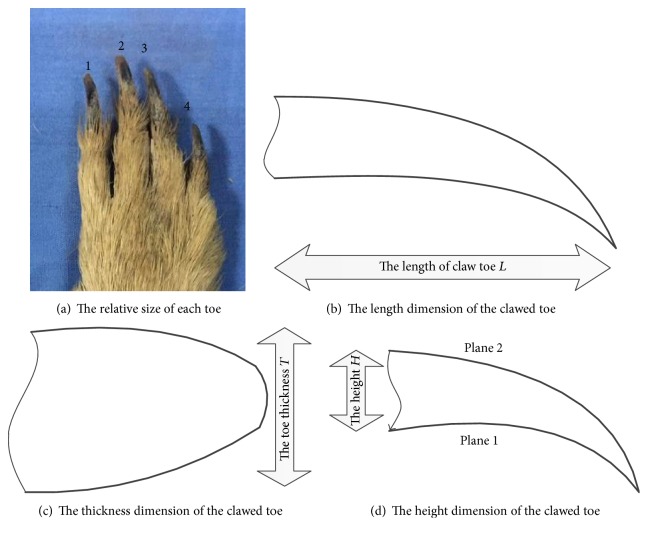
Geometrical dimensions of Himalayan marmot clawed toe.

The characteristics of the Himalayan marmot which give it great digging abilities have the potential to be developed and utilized by humans. Its body is stocky and its limbs are short and thick with stubby forelegs and sharp claws that facilitate digging; its clawed toes, which come into direct contact with the soil, have a typical digging structure. There are four clawed toes on its front feet and five on its hind feet. Its front palms are smaller than its hind palms. The main functions of the forelegs and hind legs are different. The main functions of the hind legs include supporting the marmot's body weight, increasing its speed, and playing a subsidiary supporting role in digging. The forelegs were selected as the bionic prototype for the sampler design. The claws selected for this study were removed from a Himalayan marmot body and air-dried. Characteristics of the clawed toes are shown in Figure 1. As can be seen in Figure 1(a), the second toe is the longest and the fourth toe is the shortest. As shown in Figures 1(b) and 1(c), the clawed toe can be described as a three-dimensional object, with length *L*, thickness *T*, and height *H*. The clawed toes of five adult marmots were measured.

**Figure 2:**
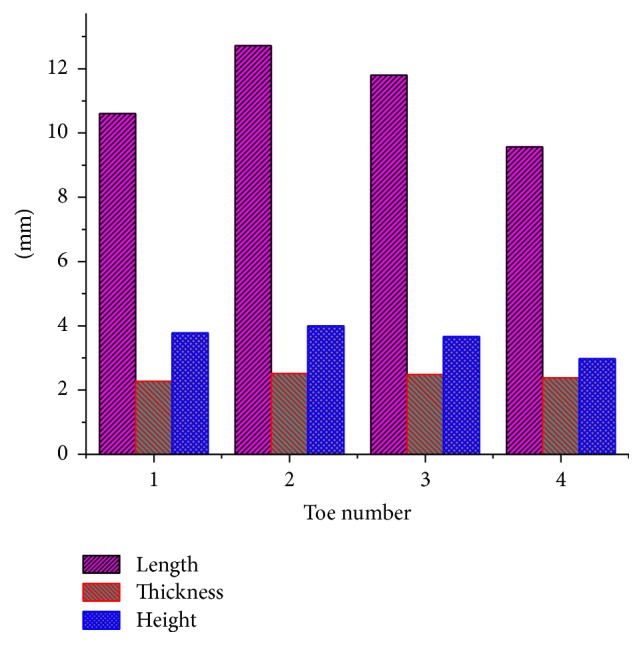
The measurements of each toe.

The macroscopic geometric parameters of the clawed toes were measured with vernier calipers. The measurements of the front clawed toes are shown in Figure 2. As it is the longest toe, the second toe is the first to touch the ground during digging. The other toes can penetrate the soil more easily and with less resistance than all the toes that penetrated the soil at the same time.

The thickness is the maximum distance between two parallel sides of the toe. Notice that there is little difference in the thickness of the toes. The second toe is the thickest. The smaller the toe thickness is, the smaller the load-acting area during digging will be and the smaller the resistance will be. However, thinner toes could result in inefficiencies during digging because soil may more easily be left between toes.

The height of the clawed toe is the distance between the horizontal planes P1 and P2, as shown in Figure 1(d). The toe heights are shown in Figure 2. Although the height differences are not great, the second toe is the highest and the fourth toe is the lowest. Through comparison and analysis of the length and height of the toes, the rule of curves of each toe can be revealed.

The second toe is longer and slightly taller and thicker than the other three toes. Therefore, the second toe of the front foot is chosen for surface morphology analysis.

### 2.2. Extracting the Clawed Toe's Point Cloud

**Figure 3:**
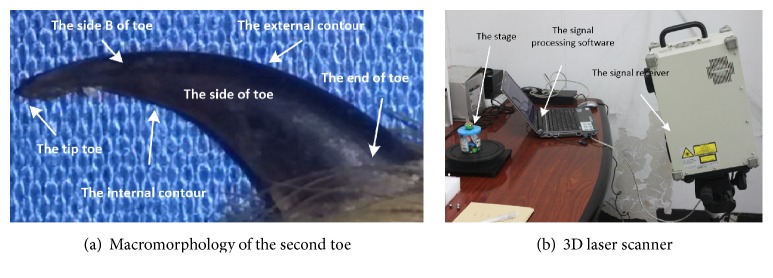
Himalayan marmot's second toe and scanning equipment.

**Figure 4:**
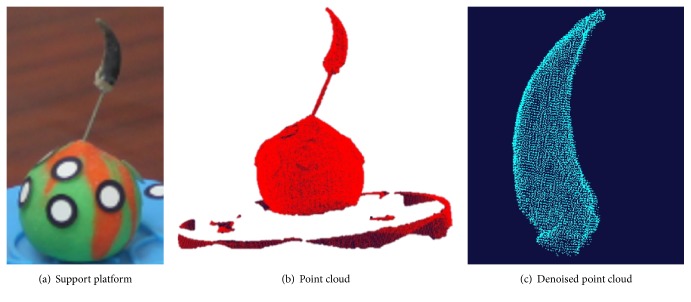
Point cloud for Himalayan marmot claw.

The Himalayan marmot's excellent ability to dig is closely related to the morphology of its clawed toes. As shown in Figure 1(a), the second toe is bigger than the other toes in all dimensions. We used a noncontact VIVID 9103d 3D laser scanner to obtain the characteristics of the claw toe point cloud, as shown in Figure 3(b). To maintain the stability of the clawed toe and guarantee accurate measurements, the support device was made of silly putty, as shown in Figure 4(a).

The process of obtaining the clawed toe's point cloud was controlled by software automatically. The stage was turned 60° between scans. The point cloud is shown in Figure 4(b) and the results after denoising and smoothing are shown in Figure 4(c).

**Figure 5:**
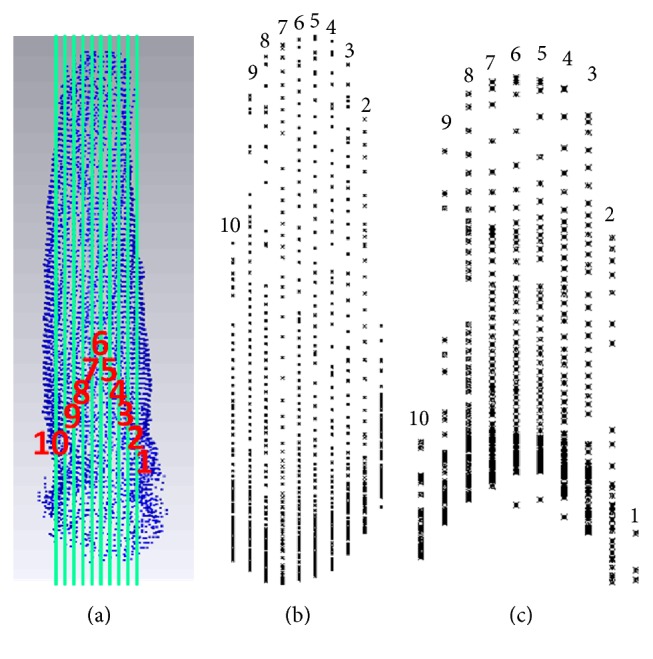
Data extraction based on point cloud data. (a) External contour; (b) external contour feature; and (c) obtained external contour feature.

Figure 5(a) shows the external contour point cloud. Using a vertical plane passing through the claw from right to left at 0.3 mm intervals, ten characteristic curves of the point clouds were extracted. The external and internal contour feature points are shown in Figures 5(b) and 5(c), respectively.

After comparison and analysis, a cubic polynomial equation was used to fit the point cloud data. The data fitting curve is evaluated by coefficient of determination *R*^2^ and residual sum of squares (SSE). The closer to zero the SSE value is, the better the fit of the curve is. *R*^2^ is close to 1, indicating a strong correlation between each pair of data.

(1) $$\begin{array}{c} f\left(\;x\;\right)=p_{1}\cdot x^{3}+p_{2}\cdot x^{2}+p_{3}\cdot x+p_{4}. \end{array}$$

**Figure 6:**
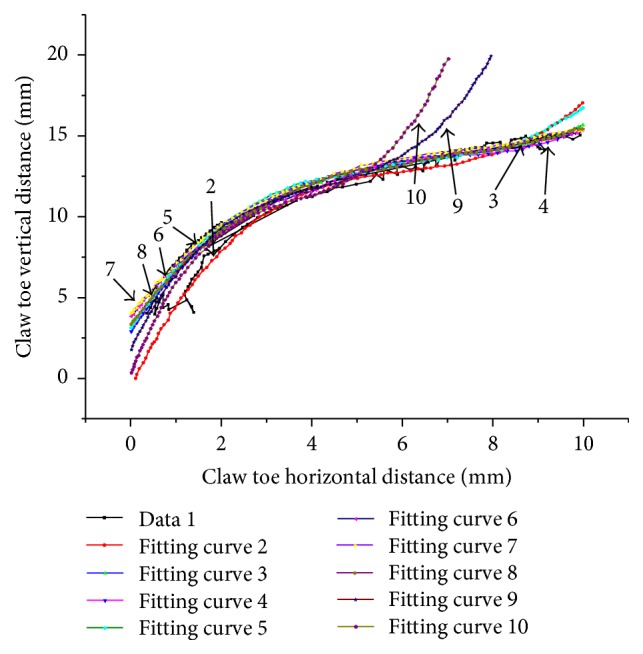
The polynomial fitting curves of the Himalayan marmot's front claw contours.

The external contour feature points were fitted using Matlab. The results of the curve fitting are shown in Figure 6.

The fitting curves of curves 2–8 were better with *x* in the range of 0.5 to 10 mm, and *R*^2^ values were all greater than 0.98. However, curves 1, 9, and 10 were not better because the sides of the claw toe contain few valid data points. These curves were removed.  Table 1 lists the other seven curve parameters.

**Figure 7:**
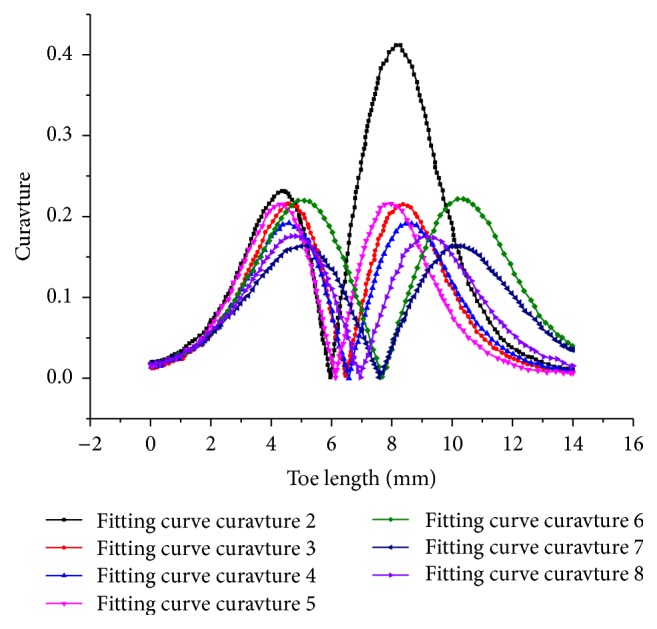
The polynomial fitting curves of the external contours of the Himalayan marmot's second front claw.

The curvature coefficient is used for determining the direction of the claw, as shown in Figure 7. All the curves showed symmetrical bimodality except curve 2. As an example, for curve 3, the curvature coefficient increased first then reduced, with a range from 0 mm to 7 mm. The same trend was apparent in the range from 7 mm to 10 mm. The minimum value of the curvature coefficient is half the length of the claw.

**Figure 8:**
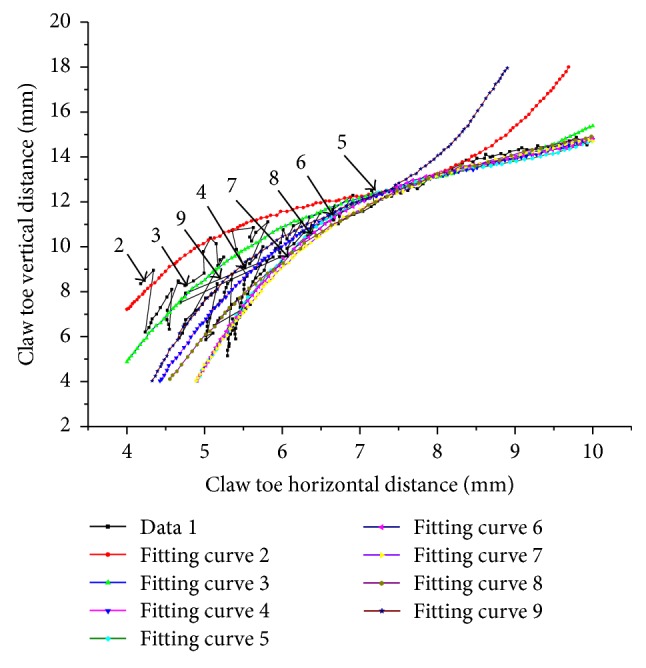
The polynomial fitting curve of the internal contours of the Himalayan marmot's second front claw.

Figure 8 shows the fitting curves of the internal contour feature points. Curves 1 and 10 are not shown because of poor fitting.  Table 2 lists the other nine curve parameters. The fit of the data is good within the range from 4 mm to 7.5 mm. Between 7.5 mm and 10 mm, curves 3–8 show a better fit than curves 2 and 9. Curves 2 and 9 are far away from the center of the claw, so the data separate. The undulatory properties were also big, so these two curves were also removed.

**Figure 9:**
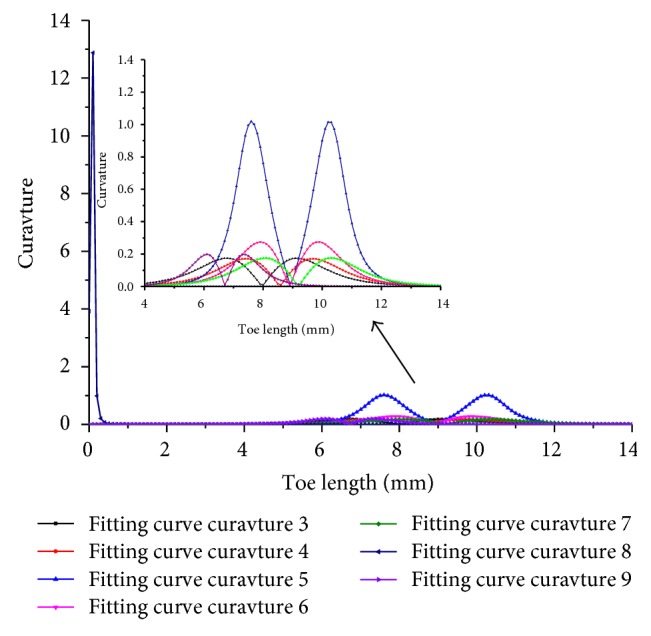
Curvature variation in the fitted curve of the inner side of the toe.

As shown in Figure 9, we calculated the curvature coefficient of fitting curves 3–9.

At effective toe lengths of 5 mm to 11 mm, the curvature coefficient of fitting curve 8 fluctuates within the range from 0 mm to 0.5 mm. The curvature coefficients of fitting curves 3–7 display symmetrical bimodality and the minimum value of the curvature coefficient is zero at toe lengths from 8 mm to 9.3 mm. The most significant changes in the curvature coefficient are shown in curve 5.

The curvature coefficient was calculated with different curves to express the degree of bending, revealing that the claw's external and internal surfaces have an axial arc, which can reduce resistance during the interaction between toe and soil.

## 3. Bionic Design and Verification

### 3.1. Bionic Design

**Figure 10:**
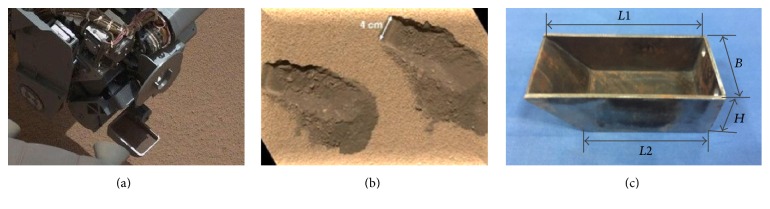
(a)* Curiosity* scoop. (b) Traces of excavation on Mars. (c) Prototype scoop.

**Figure 11:**
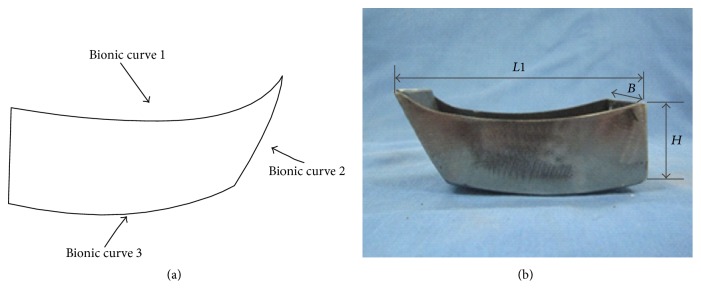
(a) Schematic diagram of the bionic sampling shovel. (b) The completed sampling shovel (*L*1 = 88.93, *B* = 45, and *H* = 30).

Based on the structure and size of the* Curiosity* sampling scoop adopted by the JPL shown in Figures 10(a) and 10(b), the sampling shovel was manufactured with the following dimensions: *L*1 = 90 mm, *B* = 45 mm, *H* = 30 mm, and *L*2 = 64 mm. At the same time, based on fitting curves of the claw, the bionic sampling scoop shown in Figure 11 was also designed.

Internal fitting curve 5 was selected as bionic curve 1 based on (1). The control equation of bionic curve 1 for the side of the bionic scale-taken sampling shovel is claw toe inner silhouette curve fitting curvature 5:

(2) $$\begin{array}{c} f\left(\;x\;\right)=0.14x^{3}-3.8x^{2}+33x-84.57;\;\left(5\leq x\leq 10\right). \end{array}$$

External fitting curves 6 and 3 were selected as bionic curves 2 and 3, respectively.

(3) fx=0.036x3−0.66x2+4.374x+3.07;1≤x≤9,fx=0.02x3−0.46x2+3.629x−3.74.

### 3.2. Experimental Verification

**Figure 12:**
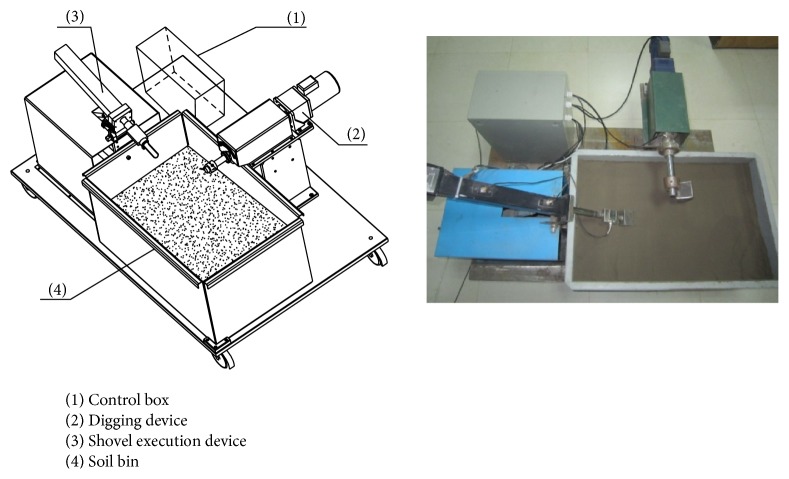
The sampling test bed.

A sampling and control system of a test bed for sampling soil was designed containing four main parts: a soil bin, a shovel device, a digging device, and a controller. The experimental apparatus is shown in Figure 12.

**Figure 13:**
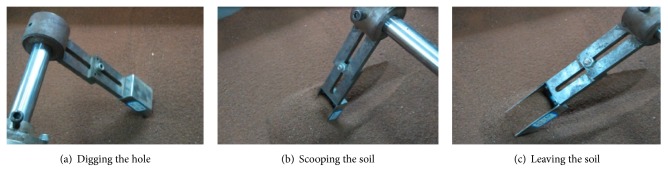
The process of digging and sampling.

The main control parameters of the shoveling device and digging device are penetration angle, speed of shoveling or digging, resistance moment, and time. For the sampling scoop, digging tests at three speeds (0.11, 0.33, and 0.56 r/min) and two penetration angles (45° and 60°) were done. The digging test is illustrated in Figure 13. The process is divided into three parts: touching the soil, rotating the sampler around its center of rotation, and leaving the soil. This sampling process was repeated three times.

**Figure 14:**
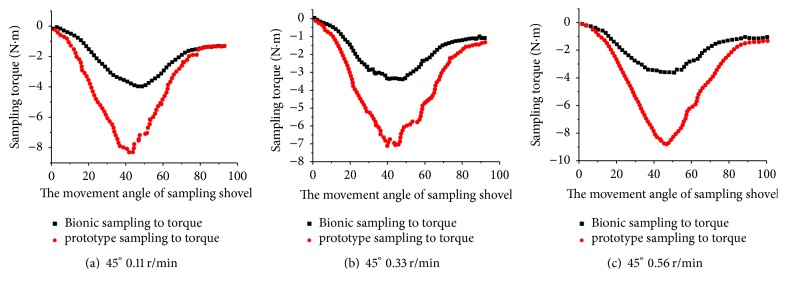
The loading torque of the bionic and prototype sampler at a penetration angle of 45°.

The torque curves of prototype sampler and bionic sampler for three different speeds and the same penetration angle (45°) are shown in Figures 14(a)–14(c). The figure shows that the bionic sampling shovel can reduce sampling resistance. At sampling speeds of 0.11 r/min, 0.33 r/min, and 0.56 r/min, bionic sampler reduces maximum torque to 55.6%, 49.6%, and 57.6%, respectively, compared with prototype sample. The torque of bionic sampling is gentle and it showed good stability in the process of repeating the test.

**Figure 15:**
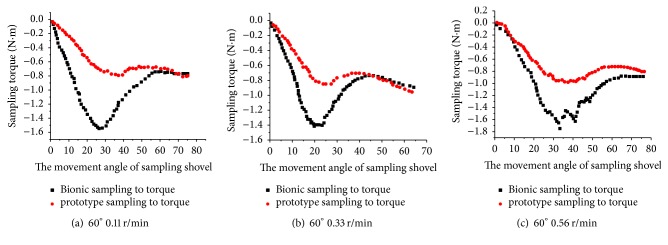
Torque values at different speeds when the penetration angle is 60°.

When the penetration angle is 60° and sampling speed is 0.11 r/min, 0.22 r/min, and 0.56 r/min, bionic sampler has less torque than prototype sample by 31.9%, 28.8%, and 27.4%, as shown in Figure 15. It is clear that the bionic sampling shovel can effectively reduce torque compared with prototype sample, and when the penetration angle is 60°, the sampling resistance is lower than when the penetration angle is 45°. This occurs because as the penetration angle increases, the hole becomes shallower and the resistance decreases.

## 4. Conclusions

We can conclude the following:

1. After measuring the toes on the front feet of the Himalayan marmot, we can see that the average length of the second toe is 12.72 mm, the thickness is 2.51 mm, and the height is 4.0 mm, which are bigger values than those for the rest of the toes. The fourth toe is the smallest. Considering the marmot's digging habits, we concluded that the second clawed toe of the front foot was the most important for digging.
2. By 3D scanning, we obtained the point cloud data for the second toe of a Himalayan marmot's front claws. We then used the polynomial fitting feature for point cloud data in Matlab and obtained fitting curves. Analyses of characteristic curves revealed that the greater the distance from the center of the toe is, the more unstable the characteristic curve fitting equation is. Analysis of the curvature coefficients revealed symmetrical bimodality; that is, the curvature showed an increase first and then reduced and then a tendency to increase after the curve was horizontal.
3. By comparison and analyses of the similarity between the Himalayan marmot's claw and the sampling scoop on* Curiosity*, we designed a prototype sampling scoop. A bionic sampling scoop was also designed based on internal and external fitting curves.
4. We analyzed the resistance moment of the bionic sample shovel and prototype sampling shovel. By comparing the torque of the bionic sampling scoop and the torque of the prototype sampling scoop, we found that the maximum torque of the bionic sampling scoop was 55.6%, 49.6%, and 55.6% less than the prototype sampling scoop at three different speeds and at a penetration angle of 45°. When the penetration angle was 60°, the maximum torque of the bionic sampling scoop was 31.9%, 28.8%, and 27.4% less than the prototype sampling scoop.

## Figures and Tables

**Table 1:** The data fitting polynomial equation coefficients and coefficients of evaluation of the outer contours of the Himalayan marmot's second front claw.

	2	3	4	5	6	7	8
*p*_1_	0.05	0.034	0.03	0.036	0.02	0.018	0.024
*p*_2_	−0.89	−0.66	−0.59	−0.66	−0.46	−0.41	−0.50
*p*_3_	5.82	4.595	4.229	4.374	3.629	3.404	3.816
*p*_4_	−0.57	2.053	2.873	3.07	3.74	3.961	3.265
SSE	9.11	7.023	5.527	3.413	4.517	2.271	3.205
*R* ^2^	0.98	0.989	0.99	0.99	0.99	0.996	0.995

**Table 2:** The data fitting polynomial equation coefficients and coefficients of evaluation of internal contours of the Himalayan marmot's second front claw.

	2	3	4	5	6	7	8	9
*p*_1_	0.15	0.08	0.09	0.14	0.12	0.09	0.05	0.31
*p*_2_	−3.1	−1.9	−2.26	−3.8	−3.1	−2.48	9.7	−6.3
*p*_3_	21.3	16.0	20.2	33.0	28.3	23.6	−1.4	43.1
*p*_4_	−38.62	−33.55	−48.52	−84.57	−73.56	−62.25	14.26	−90.2
SSE	2.662	2.32	1.38	11.25	5.63	1.448	0.47	0.16
*R* ^2^	0.97	0.99	0.988	0.975	0.987	0.99	0.999	0.997
